# Supplementary material for: Utility of Animal Models to Understand Human Alzheimer’s Disease, Using the Mastermind Research Approach to Avoid Unnecessary Further Sacrifices of Animals
Source: Int J Mol Sci. 2020 Apr 30;21(9):3158. doi: 10.3390/ijms21093158 (PMC7247586; doi:10.3390/ijms21093158)
Supplement: Supplementary file 1 [file ijms-21-03158-s001.zip › Supplementary Material 1.pdf]

Supplementary Material 1

Overview of the pathological hypothese in AD

1. Cholinergic Hypothesis

The cholinergic hypothesis was proposed by Davies and Maloney in 1976 [1]. They compared the activities of the key enzymes involved in the synthesis of the neurotransmitters in different brain regions of brains from AD patients and control and found that the activity of choline acetyltransferase in the AD brains was greatly reduced in the amygdala, hippocampus, and cortex, in which the concentration of acetylcholine (ACh) was decreased at synapses [2]. As ACh is a neurotransmitter involved in critical physiological processes such as attention, learning, memory, stress response, wakefulness and sleep, and sensory information, this was the first time that AD was described as cholinergic system failure [3,4] and cholinergic neuron damage was considered to be a critical pathological change that correlated with cognitive impairment in AD. However, not all treatments that are based upon the cholinergic hypothesis have been successful in treating the symptoms or slowing the progression of AD. Therefore, a disruption to the cholinergic system has been proposed as a consequence of AD rather than a direct cause.

2. Tau Hypothesis

The tau hypothesis is based on the loss of microtubule-stabilizing tau protein that leads to the degradation of the cytoskeleton of brain cells, and the formation of intracellular neurofibrillary tangles (NFTs), consisting of abnormally phosphorylated and aggregated tau protein [5–7], thereby destabilizing microtubules and compromising axonal transport [5–9], especially in memory-associated brain regions of AD patients [10,11]. Pathological alterations of tau were thought to be downstream events of Aβ deposition; however, tau and Aβ may act as well in parallel, enhancing each other's toxic effects and initiate the pathogenic events germane to AD [12]. Recent studies also point to soluble, diffusible tau oligomers as important drivers of tau related disease progression in the brain [13–16].

3. Amyloid Cascade Hypothesis

The amyloid cascade hypothesis was first proposed in 1991 by Selkoe and Hardy and Allsop [17,18]. They proposed that the pathological cascades in AD start with the deregulation of the APP gene, followed by Aβ deposition [17], followed by tau phosphorylation, NFT formation, and eventually neuronal death [18]. The central role of the amyloid hypothesis in AD pathogenesis has been supported by the fact that familial forms of AD are caused by overproduction of Aβ peptides with a tendency to misfold. Familial forms of AD are caused by mutations in either the APP gene, which is sequential cleaved by β-secretase (BACE1) and γ-secretases which has a length of 40 amino residues, or in the genes encoding presenilin 1 (PSEN1) or presenilin 2 (PSEN2), which are essential components of the γ-secretase complex [19]. A small fraction (around 10%) corresponds to the $A\beta_{1-42}$ variant [20], which is a highly hydrophobic protein and prone to aggregation, and the most pathogenic form. Overall, these mutations lead to the elevation of total Aβ and a higher $A\beta_{1-42}/A\beta_{1-40}$ ratio, and Aβ aggregation leading to the formation of extracellular Aβ plaques [21].

4. Reactive Gliosis and Inflammation Hypothesis

Besides Aβ and tau, reactive gliosis and neuroinflammation also seem to play an important role in AD [22,23]. Emerging genetic and transcriptomic studies showed that microglia-related pathways are important for AD risk and pathogenesis [24]. It has been shown that microglia and astrocyte cells exhibit higher activity in AD patients compared to the control group [25]. Reactive microglia and astrocytes will surround Aβ plaques and release inflammation-inducing factors, such as TNF-α, to cause immune responses. In addition, also numerous pro-inflammatory cytokines such as IL-1β, TGF-β, IL-12, and IL-18 are secreted [26].

5. Oxidative Stress and Mitochondrial Cascade Hypothesis

Oxidative stress is considered to play an important role in the pathogenesis of AD [27–29]. Cellular oxidative stress may include increased protein oxidation, protein nitration, glycol oxidation and lipid peroxidation as well as accumulation of Aβ. Moreover, Aβ can also induce oxidative stress [28]. The level of oxidative stress is closely related to the mitochondrial function in AD [30] as evidence has shown that in AD brains, the production of reactive oxygen species (ROS) increases due to mitochondrial damage [31]. Furthermore, the brains of AD patients have elevated levels of oxidative DNA damage in both nuclear and mitochondrial DNA, with the mitochondrial DNA having approximately 10-fold higher levels compared to nuclear DNA. Aged mitochondria may be the critical factor in the origin of neurodegeneration in AD [32]. Even individuals with mild cognitive impairment, the phase between normal aging and early dementia, have increased oxidative damage in their nuclear and mitochondrial brain DNA [33]. Moreover, a two hit hypothesis was introduced by Zhu et al. 2004, suggesting that both oxidative stress and mitogenic dysregulation are necessary and sufficient to cause AD [34,35].

6. Neurovascular Function Hypothesis

Alterations in cerebrovascular function are features of both cerebrovascular pathologies and neurodegenerative diseases, including AD [36]. The damage of cerebral microvasculature in AD was first reported in 1994 [37]. Making use of an AD mouse model, Thomas et al. (1996), found that Aβ can induce the constriction of the cerebral arteries [38] and impairment of neocortical microcirculation, even before the accumulation of Aβ [39]. Furthermore, dysfunction of low density lipoprotein receptor related protein-1 (LRP-1) at the BBB has been found as a possible mechanism leading to the increase of Aβ levels in the brain [40]. In 2011, Zlokovic introduced the "two-hit vascular hypothesis for Alzheimer's disease", with vascular risk factors as hit one, leading to an increase in Aβ in the brain, eventually leading to neurodegeneration and dementia [41]. Also, the role of the efflux transporter P-glycoprotein at the BBB has been related to reduced clearance of Aβ from the brain [42]. Currently, the focus is on changes in the functioning of the BBB in AD and other neurodegenerative disorders [43,44].

Reference

1. Davies, P.; Maloney, A.J.F. Selective loss of central cholinergic neurons in Alzheimer's disease. *The Lancet* **1976**, *308*, 1403.
2. Francis, P.T.; Palmer, A.M.; Snape, M.; Wilcock, G.K. The cholinergic hypothesis of Alzheimer's disease: A review of progress. *J. Neurol. Neurosurg. Psychiatry.* **1999**, *66*, 137–147.
3. Fotiou, D.; Kaltsatou, A.; Tsiptsios, D.; Nakou, M. Evaluation of the cholinergic hypothesis in Alzheimer's disease with neuropsychological methods. *Aging Clin. Exp. Res.* **2015**, *27*, 727–733.
4. Ferreira-Vieira, T.H.; Guimaraes, I.M.; Silva, F.R.; Ribeiro, F.M. Alzheimer's disease: Targeting the Cholinergic System. *Curr. Neuropharmacol.* **2016**, *14*, 101–115.
5. Querfurth, H.W.; LaFerla, F.M. Alzheimer's Disease. *N. Engl. J. Med.* **2010**, *362*, 329–344.
6. Medeiros, R.; Baglietto-Vargas, D.; LaFerla, F.M. The role of tau in Alzheimer's disease and related disorders. *CNS Neurosci. Ther.* **2011**, *17*, 514–524.
7. Morris, M.; Maeda, S.; Vossel, K.; Mucke, L. The many faces of tau. *Neuron* **2011**, *70*, 410–426.
8. Ittner, L.M.; Gotz, J. Amyloid-beta and tau--a toxic pas de deux in Alzheimer's disease. *Nat. Rev. Neurosci.* **2011**, *12*, 65–72.
9. Scheltens, P.; Blennow, K.; Breteler, M.M.B.; de Strooper, B.; Frisoni, G.B.; Salloway, S.; Van der Flier, W.M. Alzheimer's disease. *The Lancet* **2016**, *388*, 505–517.
10. Fu, H.; Rodriguez, G.A.; Herman, M.; Emrani, S.; Nahmani, E.; Barrett, G.; Figueroa, H.Y.; Goldberg, E.; Hussaini, S.A.; Duff, K.E. Tau Pathology Induces Excitatory Neuron Loss, Grid Cell Dysfunction, and Spatial Memory Deficits Reminiscent of Early Alzheimer's Disease. *Neuron* **2017**, *93*, 533–541 e5.
11. Du, X.; Wang, X.; Geng, M. Alzheimer's disease hypothesis and related therapies. *Transl. Neurodegener.* **2018**, *7*, 2.

12. Bennett, R.E.; DeVos, S.L.; Dujardin, S.; Corjuc, B.; Gor, R.; Gonzalez, J.; Roe, A.D.; Frosch, M.P.; Pitstick, R.; Carlson, G.A.; et al. Enhanced Tau Aggregation in the Presence of Amyloid beta. *Am. J. Pathol.* **2017**, *187*, 1601–1612.
13. Fa, M.; Puzzo, D.; Piacentini, R.; Staniszewski, A.; Zhang, H.; Baltrons, M.A.; Li Puma, D.D.; Chatterjee, I.; Li, J.; Saeed, F.; et al. Extracellular Tau Oligomers Produce An Immediate Impairment of LTP and Memory. *Sci. Rep.* **2016**, *6*, 19393.
14. Piacentini, R.; Li Puma, D.D.; Mainardi, M.; Lazzarino, G.; Tavazzi, B.; Arancio, O.; Grassi, C. Reduced gliotransmitter release from astrocytes mediates tau-induced synaptic dysfunction in cultured hippocampal neurons. *Glia* **2017**, *65*, 1302–1316.
15. Puzzo, D.; Piacentini, R.; Fa, M.; Gulisano, W.; Li Puma, D.D.; Staniszewski, A.; Zhang, H.; Tropea, M.R.; Cocco, S.; Palmeri, A.; et al. LTP and memory impairment caused by extracellular Abeta and Tau oligomers is APP-dependent. *Elife* **2017**, *6*, doi:10.7554/eLife.26991.
16. Reilly, P.; Winston, C.N.; Baron, K.R.; Trejo, M.; Rockenstein, E.M.; Akers, J.C.; Kfoury, N.; Diamond, M.; Masliah, E.; Rissman, R.A.; et al. Novel human neuronal tau model exhibiting neurofibrillary tangles and transcellular propagation. *Neurobiol. Dis.* **2017**, *106*, 222–234.
17. Selkoe, D.J. The molecular pathology of Alzheimer's disease. *Neuron* **1991**, *6*, 487–498.
18. Hardy, J.; Allsop, D. Amyloid deposition as the central event in the aetiology of Alzheimer's disease. *Trends Pharmacol. Sci.* **1991**, *12*, 383–388.
19. Karran, E.; Mercken, M.; De Strooper, B. The amyloid cascade hypothesis for Alzheimer's disease: An appraisal for the development of therapeutics. *Nat. Rev. Drug Discov.* **2011**, *10*, 698–712.
20. Jarrett, J.T.; Berger, E.P.; Lansbury, P.T. The carboxy terminus of the .beta. amyloid protein is critical for the seeding of amyloid formation: Implications for the pathogenesis of Alzheimer's disease. *Biochemistry* **1993**, *32*, 4693–4697.
21. Pimplikar, S.W. Reassessing the amyloid cascade hypothesis of Alzheimer's disease. *Int. J. Biochem. Cell Biol.* **2009**, *41*, 1261–1268.
22. Calsolaro, V.; Edison, P. Neuroinflammation in Alzheimer's disease: Current evidence and future directions. *Alzheimers Dement.* **2016**, *12*, 719–732.
23. Butterfield, D.A.; Halliwell, B. Oxidative stress, dysfunctional glucose metabolism and Alzheimer disease. *Nat. Rev. Neurosci.* **2019**, *20*, 148–160.
24. Bagyinszky, E.; Giau, V.V.; Shim, K.; Suk, K.; An, S.S.A.; Kim, S. Role of inflammatory molecules in the Alzheimer's disease progression and diagnosis. *J. Neurol. Sci.* **2017**, *376*, 242–254.
25. Santos, L.E.; Beckman, D.; Ferreira, S.T. Microglial dysfunction connects depression and Alzheimer's disease. *Brain Behav. Immun.* **2016**, *55*, 151–165.
26. Acosta, C.; Anderson, H.D.; Anderson, C.M. Astrocyte dysfunction in Alzheimer disease. *J. Neurosci. Res.* **2017**, *95*, 2430–2447.
27. Markesbery, W.R. Oxidative Stress Hypothesis in Alzheimer's Disease. *Free Radic. Biol. Med.* **1997**, *23*, 134–147.
28. Padurariu, M.; Ciobica, A.; Lefter, R.; Serban, I.L.; Stefanescu, C.; Chirita, R. The oxidative stress hypothesis in Alzheimer's disease. *Psychiatria Danubina* **2013**, *25*, 401–409.
29. Kamat, P.K.; Kalani, A.; Rai, S.; Swarnkar, S.; Tota, S.; Nath, C.; Tyagi, N. Mechanism of Oxidative Stress and Synapse Dysfunction in the Pathogenesis of Alzheimer's Disease: Understanding the Therapeutics Strategies. *Mol. Neurobiol.* **2016**, *53*, 648–661.
30. Swerdlow, R.H.; Khan, S.M. A "mitochondrial cascade hypothesis" for sporadic Alzheimer's disease. *Med. Hypotheses* **2004**, *63*, 8–20.
31. Uttara, B.; Singh, A.V.; Zamboni, P.; Mahajan, R.T. Oxidative stress and neurodegenerative diseases: A review of upstream and downstream antioxidant therapeutic options. *Curr. Neuropharmacol.* **2009**, *7*, 65–74.
32. Santos, R.X.; Correia, S.C.; Zhu, X.; Smith, M.A.; Moreira, P.I.; Castellani, R.J.; Nunomura, A.; Perry, G. Mitochondrial DNA oxidative damage and repair in aging and Alzheimer's disease. *Antioxid. Redox Signal.* **2013**, *18*, 2444–2457.
33. Cenini, G.; Voos, W. Mitochondria as Potential Targets in Alzheimer Disease Therapy: An Update. *Front. Pharmacol.* **2019**, *10*, 902.
34. Zhu, X.; Raina, A.K.; Perry, G.; Smith, M.A. Alzheimer's disease: The two-hit hypothesis. *The Lancet Neurology* **2004**, *3*, 219–226.

35. Zhu, X.; Lee, H.G.; Perry, G.; Smith, M.A. Alzheimer disease, the two-hit hypothesis: An update. *Biochim. Biophys. Acta* **2007**, 1772, 494–502.
36. Iadecola, C. Neurovascular regulation in the normal brain and in Alzheimer's disease. *Nat. Rev. Neurosci.* **2004**, *5*, 347–360.
37. Buée, L.; Hof, P.R.; Bouras, C.; Delacourte, A.; Perl, D.P.; Morrison, J.H.; Fillit, H.M. Pathological alterations of the cerebral microvasculature in Alzheimer's disease and related dementing disorders. *Acta Neuropathologica* **1994**, *87*, 469–480.
38. Thomas, T.; Thomas, G.; McLendon, C.; Sutton, T.; Mullan, M. β-Amyloid-mediated vasoactivity and vascular endothelial damage. *Nature* **1996**, *380*, 168–171.
39. Iadecola, C.; Zhang, F.; Niwa, K.; Eckman, C.; Turner, S.K.; Fischer, E.; Younkin, S.; Borchelt, D.R.; Hsiao, K.K.; Carlson, G.A. SOD1 rescues cerebral endothelial dysfunction in mice overexpressing amyloid precursor protein. *Nature Neurosci.* **1999**, *2*, 157–161.
40. Jaeger, L.B.; Dohgu, S.; Hwang, M.C.; Farr, S.A.; Murphy, M.P.; Fleegal-DeMotta, M.A.; Lynch, J.L.; Robinson, S.M.; Niehoff, M.L.; Johnson, S.N.; et al. Testing the neurovascular hypothesis of Alzheimer's disease: LRP-1 antisense reduces blood-brain barrier clearance, increases brain levels of amyloid-beta protein, and impairs cognition. *J. Alzheimers Dis.* **2009**, *17*, 553–570.
41. Zlokovic, B.V. Neurovascular pathways to neurodegeneration in Alzheimer's disease and other disorders. *Nat. Rev. Neurosci.* **2011**, *12*, 723–738.
42. Erdo, F.; Krajcsi, P. Age-Related Functional and Expressional Changes in Efflux Pathways at the Blood-Brain Barrier. *Front. Aging Neurosci.* **2019**, *11*, 196.
43. Nation, D.A.; Sweeney, M.D.; Montagne, A.; Sagare, A.P.; D'Orazio, L.M.; Pachicano, M.; Sepehrband, F.; Nelson, A.R.; Buennagel, D.P.; Harrington, M.G.; et al. Blood-brain barrier breakdown is an early biomarker of human cognitive dysfunction. *Nat. Med.* **2019**, *25*, 270–276.
44. Sweeney, M.D.; Sagare, A.P.; Zlokovic, B.V. Blood-brain barrier breakdown in Alzheimer disease and other neurodegenerative disorders. *Nat. Rev. Neurol.* **2018**, *14*, 133–150.
