# Supplementary material for: Utility of Animal Models to Understand Human Alzheimer’s Disease, Using the Mastermind Research Approach to Avoid Unnecessary Further Sacrifices of Animals
Source: Int J Mol Sci. 2020 Apr 30;21(9):3158. doi: 10.3390/ijms21093158 (PMC7247586; doi:10.3390/ijms21093158)
Supplement: Supplementary file 1 [file ijms-21-03158-s001.zip › Supplementary Material 2.pdf]

Supplementary Material 2

Table S1. Overview of frequently studied biomarkers belonging to the different processes linked to AD, in plasma, CSF and brain of AD patients and the most frequently used rat and mouse models of AD.

Brain

**Biomarker**	**Aβ_{1-40}**	**Aβ_{1-42}**	**Aβ_{1-42}/ Aβ_{1-40}**	**P-tau**	**T-tau**	**NfL**	**Neuro-granin**	**SNAP-25**	**GFAP**	**YKL-40**
**Human**										
**AD Patient**	[1]	[1]		[2]			[3]	[4,5]	[6]<br>[8] **(cerebellum)**	[7]
**Transgenic mice**										
**PDAPP**	[9]									
**Tg2576**	[10–12]	[10,12,13]								
**APP/PS1**	[11,14–16]	[14,15]							[14]	
**3xTg-AD**	[17,18]	[17,18]		[18]					[19]	
**APPPS1**	[20–22]	[20–22]							[20]	
**APP23**	[11,21,23]	[21,23,24]		[25]						
**Transgenic rat**										
**McGill-R-Thy1-APP**	[26]	[26,27]							[27]	
**Tg478/Tg1116/Tg11587 triple homozygous**	[28]	[28,29]							[28]	
**TgF344-AD**	[30]	[30]		[30,31]					[30]	
**Chemical induced non transgenic rat model**										
**Ferrous amyloid buthionine (FAB) rat**		[32]		[32]					[32]	
**Aβ42 oligomer (AβO) induced rat**		[33]		[33]						

Changes with respect to the control group versus AD patients or young versus old animals:

= Increase =Decrease = No change = No data

CSF

**Biomarker**	**Aβ_{1-40}**	**Aβ_{1-42}**	**Aβ_{1-42}/ Aβ_{1-40}**	**P-tau**	**T-tau**	**NfL**	**Neuro-granin**	**SNAP-25**	**GFAP**	**YKL-40**
**AD patients**	[34–39] Control 894 ± 759pM AD 981 ± 409pM [34]; Control 1415.5±482.0pM MCI 1579.9±692.1pM AD	[5,35–50] Control 191.6±75.5pm MCI 201.2±111.8pm AD 122.7±56.5pm [35]; Control 139 ± 202pm AD 73.6 ±	[37,52–54] Control 554.0±195.1 pg/ml SCD 588.8±253.4pg/ml MCI 470.1±232.3	[5,39,43–50,55] CUAB- 17.5±5.3pg/ml CUAB+ 28.5±12 pg/ml MCIAB- 16.9±6.4 pg/ml MCIAB+ 32.2±12.5pg/ml ADAB+ 36.3±16.3 pg/ml [39]	[5,36,38,39,42–50,55,56] Control 168pg/ml (IQR 132-228) AD 698pg/ml (IQR 491-1013)	[39,56,57] Control 138±31 ng/l AD 346±176 ng/l [57]; Control 595 pg/ml (IQR 430–831) AD 1405pg/ml (IQR 942–	[36,43,46,50,58,59] Control 7.8(1.5-22.8) pg/ml MCI 33.8 (19.0–73.3)pg/ml AD 36.6 (22.4–51.9)pg/ml	[5,49,60,61] Exact data are displayed in figures.	[56,62] Control 0.665ng/ml (IQR 0.409–0.978) AD 1.081ng/ml (IQR 0.534–	[7,45,56,58,63,64] Control 145 ng/ml (IQR 115-161) AD 240 ng/ml (IQR 176-193) [56]; CDR(clinical

	1387.9±518.6pM [35]; Control 4688.5±1650.0 pg/ml SCD 4966.5±1750.5pg/ml MCI 4765.3±pg/ml AD 4387.2±1761.pg/ml Pa. CUAB- 18.2±5.2 ng/ml [37]; CUAB+ 19.5±5.9ng/ml MCIAB- 17.3±5.7ng/ml MCIAB+ 17.8±5.0ng/ml ADAB+ 17.9±6.2 ng/ml [39]; Control 4531 (3216–5703) pg/ml MCI 5093 (4290–6999) pg/ml AD 4917 (4143–5460) pg/ml High Tau 6039 (5104–8380) pg/ml [36]	41.8pm [34]; Control 554.0±195.1pg/ml SCD 588.8±253.4pg/ml MCI 470.1±232.3 pg/ml AD 289.5±103.8pg/ml [37]; CUAB- 1665±596 pg/ml CUAB+ 819±303 pg/ml MCIAB- 1572±605 pg/ml MCIAB+ 706±256pg/ml ADAB+ 671±315 pg/ml [39]; Control 341 (205–461)pg/ml MCI 192 (151–254) pg/ml AD 170 (139–235)pg/ml High Tau 200 (141–228)pg/ml[36] [51] (familial AD)	pg/ml AD 289.5± 103.8pg/ml [37];	[51] (familial AD)	[56]; Control 215 (134–295) pg/ml MCI 434 (331–580) pg/ml AD 506 (423–585) pg/ml High Tau 813 (703–1041) pg/ml [36]	1730) [56]; CUAB- 918 ±490 pg/ml CUAB+ 1216±842 pg/ml MCIAB- 1648±1517 pg/ml MCIAB+ 1531±1195pg/ml ADAB+ 2002±1835 pg/ml [39]	High Tau 102.3 (68.9–149.4)pg/ml [36]; Control 291 (MSD 251-438)pg/ml AD 620 (MSD 521-818)pg/ml [59]; Control 324 (IQR 191–468)pg/ml MCI 455 (IQR 267–657)pg/ml AD 471 (IQR 347–675)pg/ml [50]		1.422) [56]; Control.M 2.08±0.77ng/ml Control.F 2.27±0.94ng/ml AD.M 3.05±1.01ng/ml AD.F 2.73±0.96ng/ml [62]	dementia rating)0 282.1±6.7ng/ml CDR0.5 358.9±16.9ng/ml CDR1 351.7±22,6ng/ml [63]; Anto. Control 260.5±71.6ng/ml pre-AD 330±120.1ng/ml Prod-AD 364.1±81.9ng/ml [45]
**Transgenic mice**										
**PDAPP**										
**Tg2576**	[12] 6month 12000pM(6month) 8000pM(23month)	[12]1500pM (6month) 800pM(23month)								
**APP/PS1**		[65] 8.6 ng/ml (6 months) 7 ng/ml (9months								

		)								
**3xTg-AD**	[66] 2200pg/ml (3month) 600pg/ml (12month)	[66] 1500pg/ml (3month) 800pg/ml (12month)								
**APPPS1**	[21] 3800pg/ml (6month) 2200pg/ml (18month)	[21] 2200pg/ml (6month) 1000pg/ml (18month)	[21] 6(3month) 3.7 (18month)		[21] 1100pg/ml (6month) 2700pg/ml (18month)	[67] 500pg/ml (3month) 3600pg/ml (12month) 12700pg/ml (18month)				
**APP23**	[21,23] 37800pg/ml (16month) 27500pg/ml (30month)	[21,23] 5000pg/ml (16month) 2200pg/ml (30month)	[23] 0.16 (3month) 0.1(25month)		[21] 300pg/ml (12month) 1350pg/ml (24month)					
	**Transgenic rats**									
**McGill-R-Thy1-APP**		[68] 2000pg/ml (10month) 1400pg/ml (17month)								
**Tg478/Tg1116/Tg11587 triple homozygous**										
**TgF344-AD**										
	**Chemical induced non transgenic rat models**									
**Ferrous amyloid buthionine (FAB) rat**				[32] 429±175 pg/ml (after treatment) N/A in control						
**Aβ42 oligomer (AβO) induced rat**										

Changes with respect to the control group versus AD patients or young versus old animals:

= Increase =Decrease = No change = No data

Blood(plasma)										
**Biomarker**	**$A\beta_{1-40}$**	**$A\beta_{1-42}$**	**$A\beta_{1-42}$/ $A\beta_{1-40}$**	**P-tau**	**T-tau**	**NfL**	**Neuro-granin**	**SNAP-25**	**GFAP**	**YKL-40**
**AD patients**	[1,35,69,70] Control 64.8±17.0pm MCI 61.1±16.3pm AD 60.2±13.1pm [35]; Control 157.8±31.9 pg/ml MCI 166.9±37.6pg /ml AD 172.2±40.9pg /ml [70]	[1,35,69,70] Control 25.4±27.6p m MCI 20.6±11.9p m AD 21.6±19.1p m [35]; Control 34.9±9.4 pg/ml MCI 33.6±11.6p g/ml AD 34.6±10.7p g/m [70]	[71,72] Ve. AB- 49.5±6,8 1 AB+ 43.5±5.5 1 [71]	[73] Exact data are displa yed in figure s.	[36,39] CUAB- 16.6±4.7 pg/ml CUAB+ 17.9±5.4 pg/ml MCIAB- 18.7±6.1 pg/ml MCIAB+ 19.1±5.2pg /ml ADAB+ 16.7±6.0pg /m [39]; Control 11.7 (5.7–26.7)AU MCI 16.7 (10.3–26.2)AU AD 16.5 (9.7–24.3)AU High Tau 13.4 (6.6–22.2)AU [36]	[38,39,48 ,74,75] CUAB- 21.0±11. 8 pg/ml CUAB+ 29.1±59. 6 pg/ml MCIAB- 28.3±28. 4 pg/ml MCIAB+ 29.0±17. 9pg/ml ADAB+ 43.8±28. 7pg/ml [39]	[36,59] Control 1.29 (0.15–3.78) ng/ml MCI 0.14 (0.05–1.01) ng/ml AD 0.88 (0.03–2.91) ng/ml High Tau 0.11 (0.02–1.91) ng/ml [36] ; Control 47451(MS D 21904-90320)pg/ ml AD 36525 (MSD 25324-57715)pg/ ml [59]		[76] (serum) Control 157 (IQR 126-218)pg/ml AD 376 (IQR 294-537)pg/ml	[63,77] CDR(cli nical dementi a rating)0 62.5±3.4 ng/ml CDR0.5 81.1±8ng /ml CDR1 91.9±15n g/ml [63]; Control 84±84ng/ ml AD 133±110 ng/ml [77]
	[37,39] Control 276.7±66.1 pg/ml SCD 276.9±76.1pg /ml MCI 287.6±77.0 pg/ml AD 244.3± 105.8pg/ml [37] ; CUAB- 482±63.3 pg/ml CUAB+ 479±67.5 pg/ml MCIAB- 495±83.2 pg/ml MCIAB+ 492±75.4pg/ ml ADAB+ 380±131.7pg/ ml [39]	[37,39] Control 16.9±5.2 pg/ml SCD 18.8±5.4pg /ml MCI 18.8±6.1 pg/ml AD 13.2± 7.3pg/ml [37]; CUAB- 32.8±4.9 pg/ml CUAB+ 29.6±4.3 pg/ml MCIAB- 33.1±5.2 pg/ml MCIAB+ 30.3±4.5pg /ml ADAB+ 23.3±8.2pg /m [39]			[44,73]					
		[51] (familial AD)			[47] Increase/n o					

					Change (depending on included study)					
	Transgenic mice									
**PDAPP**										
**Tg2576**	[12] 4000pM (6month) 2000pM(23month)	[12] 500pM (6month) 300pM (23month)								
**APP/PS1**	[15] 620pg/ml (3month) 400pg/ml (8month)	[15] 160pg/ml (3month) 50pg/ml (8month)								
	[16] Decrease/Increase (sex dependent) 750pg/ml (3month M) 500pg/ml (15month M) 750pg/ml (3month F) 1500pg/ml (15month F)	[16] 1200pg/ml (3month M/F) 1100-1400 pg/ml (15month M/F)								
**3xTg-AD**	[66] Age dependent: first up then down 70pg/ml (5month) 190pg/ml (9month) 20pg/ml (12month)	[66] Age dependent: first up then down 25pg/ml (5month) 70pg/ml (9month) 20pg/ml (12month)		[78] (in some cases no change, depending on antibody)	[78]					
**APPPS1**	[22] 0.15pg/ml/ug protein (3.5month) 0.132 (8month)	[22] 8.5pg/ml/ ug protein (3.5month) 3 (8month)	[22] 62 (3.5month) 24(8month)			[67] 15pg/ml (1.5month) 57pg/ml (7.5month)				
**APP23**										
	Transgenic rat									
**McGill-R-Thy1-APP**										
**Tg478/Tg1116/Tg11587 triple homozygous**										
**TgF344-AD**										
	Chemical induced non transgenic rat model									
**Ferrous amyloid buthionine (FAB) rat**										
**Aβ42 oligomer (AβO) induced rat**										

Changes with respect to the control group versus AD patients or young versus old animals:

= Increase =Decrease = No change = Inconsistent = No data

References

1. Roher, A.E.; Esh, C.L.; Kokjohn, T.A.; Castaño, E.M.; Van Vickle, G.D.; Kalback, W.M.; Patton, R.L.; Luehrs, D.C.; Daugs, I.D.; Kuo, Y.-M.; et al. Amyloid beta peptides in human plasma and tissues and their significance for Alzheimer's disease. *Alzheimers Dement.* **2009**, *5*, 18–29.
2. Gao, Y.; Tan, L.; Yu, J.T.; Tan, L. Tau in Alzheimer's Disease: Mechanisms and Therapeutic Strategies. *Curr. Alzheimer Res.* **2018**, *15*, 283–300.
3. Kvartsberg, H.; Lashley, T.; Murray, C.E.; Brinkmalm, G.; Cullen, N.C.; Hoglund, K.; Zetterberg, H.; Blennow, K.; Portelius, E. The intact postsynaptic protein neurogranin is reduced in brain tissue from patients with familial and sporadic Alzheimer's disease. *Acta Neuropathol.* **2019**, *137*, 89–102.
4. Shimohama, S.; Kamiya, S.; Taniguchi, T.; Akagawa, K.; Kimura, J. Differential Involvement of Synaptic Vesicle and Presynaptic Plasma Membrane Proteins in Alzheimer's Disease. *Biochem. Biophys. Res. Commun.* **1997**, *236*, 239–242.
5. Ohrfelt, A.; Brinkmalm, A.; Dumurgier, J.; Zetterberg, H.; Bouaziz-Amar, E.; Hugon, J.; Paquet, C.; Blennow, K. A Novel ELISA for the Measurement of Cerebrospinal Fluid SNAP-25 in Patients with Alzheimer's Disease. *Neuroscience* **2019**, *420*, 136–144.
6. Barroeta-Espar, I.; Weinstock, L.D.; Perez-Nievas, B.G.; Meltzer, A.C.; Siao Tick Chong, M.; Amaral, A.C.; Murray, M.E.; Moulder, K.L.; Morris, J.C.; Cairns, N.J.; et al. Distinct cytokine profiles in human brains resilient to Alzheimer's pathology. *Neurobiol. Dis.* **2019**, *121*, 327–337.
7. Llorens, F.; Thune, K.; Tahir, W.; Kanata, E.; Diaz-Lucena, D.; Xanthopoulos, K.; Kovatsi, E.; Pleschka, C.; Garcia-Esparcia, P.; Schmitz, M.; et al. YKL-40 in the brain and cerebrospinal fluid of neurodegenerative dementias. *Mol. Neurodegener.* **2017**, *12*, 83.
8. Singh-Bains, M.K.; Linke, V.; Austria, M.D.R.; Tan, A.Y.S.; Scotter, E.L.; Mehrabi, N.F.; Faull, R.L.M.; Dragunow, M. Altered microglia and neurovasculature in the Alzheimer's disease cerebellum. *Neurobiol. Dis.* **2019**, *132*, 104589.
9. Games, D.; Adams, D.; Alessandrini, R.; Barbour, R.; Borthelette, P.; Blackwell, C.; Carr, T.; Clemens, J.; Donaldson, T.; Gillespie, F.; et al. Alzheimer-type neuropathology in transgenic mice overexpressing V717F β-amyloid precursor protein. *Nature* **1995**, *373*, 523–527.
10. Hsiao, K.; Chapman, P.; Nilsen, S.; Eckman, C.; Harigaya, Y.; Younkin, S.; Yang, F.; Cole, G. Correlative Memory Deficits, Aβ Elevation, and Amyloid Plaques in Transgenic Mice. *Science* **1996**, *274*, 99–103.
11. Snellman, A.; Lopez-Picon, F.R.; Rokka, J.; Salmona, M.; Forloni, G.; Scheinin, M.; Solin, O.; Rinne, J.O.; Haaparanta-Solin, M. Longitudinal amyloid imaging in mouse brain with 11C-PIB: Comparison of APP23, Tg2576, and APPswe-PS1dE9 mouse models of Alzheimer disease. *J. Nucl. Med.* **2013**, *54*, 1434–1441.
12. Kawarabayashi, T.; Younkin, L.H.; Saido, T.C.; Shoji, M.; Ashe, K.H.; Younkin, S.G. Age-dependent changes in brain, CSF, and plasma amyloid (beta) protein in the Tg2576 transgenic mouse model of Alzheimer's disease. *J. Neurosci.* **2001**, *21*, 372–381.
13. Jacobsen, J.S.; Wu, C.-C.; Redwine, J.M.; Comery, T.A.; Arias, R.; Bowlby, M.; Martone, R.; Morrison, J.H.; Pangalos, M.N.; Reinhart, P.H.; et al. Early-onset behavioral and synaptic deficits in a mouse model of Alzheimer's disease. *Proc. Natl. Acad. Sci. USA* **2006**, *103*, 5161–5166.
14. Minkeviciene, R.; Ihalainen, J.; Malm, T.; Matilainen, O.; Keksa-Goldsteine, V.; Goldsteins, G.; Iivonen, H.; Leguit, N.; Glennon, J.; Koistinaho, J.; et al. Age-related decrease in stimulated glutamate release and vesicular glutamate transporters in APP/PS1 transgenic and wild-type mice. *J. Neurochem.* **2008**, *105*, 584–594.
15. Izco, M.; Martinez, P.; Corrales, A.; Fandos, N.; Garcia, S.; Insua, D.; Montanes, M.; Perez-Grijalba, V.; Rueda, N.; Vidal, V.; et al. Changes in the brain and plasma Abeta peptide levels with age and its relationship with cognitive impairment in the APPswe/PS1dE9 mouse model of Alzheimer's disease. *Neuroscience* **2014**, *263*, 269–279.

16. Ordonez-Gutierrez, L.; Anton, M.; Wandosell, F. Peripheral amyloid levels present gender differences associated with aging in AbetaPP/PS1 mice. *J. Alzheimers Dis.* **2015**, *44*, 1063–1068.
17. Oddo, S.; Caccamo, A.; Shepherd, J.D.; Murphy, M.P.; Golde, T.E.; Kayed, R.; Metherate, R.; Mattson, M.P.; Akbari, Y.; LaFerla, F.M. Triple-Transgenic Model of Alzheimer's Disease with Plaques and Tangles. *Neuron* **2003**, *39*, 409–421.
18. Belfiore, R.; Rodin, A.; Ferreira, E.; Velazquez, R.; Branca, C.; Caccamo, A.; Oddo, S. Temporal and regional progression of Alzheimer's disease-like pathology in 3xTg-AD mice. *Aging Cell* **2019**, *18*, e12873.
19. Caruso, D.; Barron, A.M.; Brown, M.A.; Abbiati, F.; Carrero, P.; Pike, C.J.; Garcia-Segura, L.M.; Melcangi, R.C. Age-related changes in neuroactive steroid levels in 3xTg-AD mice. *Neurobiol. Aging* **2013**, *34*, 1080–1089.
20. Radde, R.; Bolmont, T.; Kaeser, S.A.; Coomaraswamy, J.; Lindau, D.; Stoltze, L.; Calhoun, M.E.; Jaggi, F.; Wolburg, H.; Gengler, S.; et al. Abeta42-driven cerebral amyloidosis in transgenic mice reveals early and robust pathology. *EMBO Rep.* **2006**, *7*, 940–946.
21. Maia, L.F.; Kaeser, S.A.; Reichwald, J.; Hruscha, M.; Martus, P.; Staufenbiel, M.; Jucker, M. Changes in Amyloid-β and Tau in the Cerebrospinal Fluid of Transgenic Mice Overexpressing Amyloid Precursor Protein. *Sci. Transl. Med.* **2013**, *5*, doi:10.1126/scitranslmed.3006446.
22. Harach, T.; Marungruang, N.; Duthilleul, N.; Cheatham, V.; Mc Coy, K.D.; Frisoni, G.; Neher, J.J.; Fak, F.; Jucker, M.; Lasser, T.; et al. Reduction of Abeta amyloid pathology in APPPS1 transgenic mice in the absence of gut microbiota. *Sci. Rep.* **2017**, *7*, 41802.
23. Maia, L.F.; Kaeser, S.A.; Reichwald, J.; Lambert, M.; Obermuller, U.; Schelle, J.; Odenthal, J.; Martus, P.; Staufenbiel, M.; Jucker, M. Increased CSF Abeta during the very early phase of cerebral Abeta deposition in mouse models. *EMBO Mol. Med.* **2015**, *7*, 895–903.
24. Van Dam, D.; D'Hooge, R.; Staufenbiel, M.; Van Ginneken, C.; Van Meir, F.; De Deyn, P.P. Age-dependent cognitive decline in the APP23 model precedes amyloid deposition. *Eur. J. Neurosci.* **2003**, *17*, 388–396.
25. Sturchler-Pierrat, C.; Abramowski, D.; Duke, M.; Wiederhold, K.-H.; Mistl, C.; Rothacher, S.; Ledermann, B.; Bürki, K.; Frey, P.; Paganetti, P.A.; et al. Two amyloid precursor protein transgenic mouse models with Alzheimer disease-like pathology. *Proc. Natl. Acad. Sci. USA* **1997**, *94*, 13287–13292.
26. Iulita, M.F.; Allard, S.; Richter, L.; Munter, L.M.; Ducatenzeiler, A.; Weise, C.; Do Carmo, S.; Klein, W.L.; Multhaup, G.; Cuello, A.C. Intracellular Abeta pathology and early cognitive impairments in a transgenic rat overexpressing human amyloid precursor protein: A multidimensional study. *Acta Neuropathol. Commun.* **2014**, *2*, 61.
27. Hanzel, C.E.; Pichet-Binette, A.; Pimentel, L.S.; Iulita, M.F.; Allard, S.; Ducatenzeiler, A.; Do Carmo, S.; Cuello, A.C. Neuronal driven pre-plaque inflammation in a transgenic rat model of Alzheimer's disease. *Neurobiol. Aging* **2014**, *35*, 2249–2262.
28. Liu, L.; Orozco, I.J.; Planel, E.; Wen, Y.; Bretteville, A.; Krishnamurthy, P.; Wang, L.; Herman, M.; Figueroa, H.; Yu, W.H.; et al. A transgenic rat that develops Alzheimer's disease-like amyloid pathology, deficits in synaptic plasticity and cognitive impairment. *Neurobiol. Dis.* **2008**, *31*, 46–57.
29. Flood, D.G.; Lin, Y.G.; Lang, D.M.; Trusko, S.P.; Hirsch, J.D.; Savage, M.J.; Scott, R.W.; Howland, D.S. A transgenic rat model of Alzheimer's disease with extracellular Abeta deposition. *Neurobiol. Aging* **2009**, *30*, 1078–1090.
30. Cohen, R.M.; Rezai-Zadeh, K.; Weitz, T.M.; Rentsendorj, A.; Gate, D.; Spivak, I.; Bholat, Y.; Vasilevko, V.; Glabe, C.G.; Breunig, J.J.; et al. A transgenic Alzheimer rat with plaques, tau pathology, behavioral impairment, oligomeric abeta, and frank neuronal loss. *J. Neurosci.* **2013**, *33*, 6245–6256.
31. Rorabaugh, J.M.; Chalermpalanupap, T.; Botz-Zapp, C.A.; Fu, V.M.; Lembeck, N.A.; Cohen, R.M.; Weinshenker, D. Chemogenetic locus coeruleus activation restores reversal learning in a rat model of Alzheimer's disease. *Brain* **2017**, *140*, 3023–3038.
32. Lecanu, L.; Greeson, J.; Papadopoulos, V. Beta-amyloid and oxidative stress jointly induce neuronal death, amyloid deposits, gliosis, and memory impairment in the rat brain. *Pharmacology* **2006**, *76*, 19–33.
33. Forny-Germano, L.; Lyra e Silva, N.M.; Batista, A.F.; Brito-Moreira, J.; Gralle, M.; Boehnke, S.E.; Coe, B.C.; Lablans, A.; Marques, S.A.; Martinez, A.M.; et al. Alzheimer's disease-like pathology induced by amyloid-beta oligomers in nonhuman primates. *J. Neurosci.* **2014**, *34*, 13629–13643.
34. Fukuyama, R.; Mizuno, T.; Mori, S.; Nakajima, K.; Fushiki, S.; Yanagisawa, K. Age-dependent change in the levels of Abeta40 and Abeta42 in cerebrospinal fluid from control subjects, and a decrease in the ratio

of Abeta42 to Abeta40 level in cerebrospinal fluid from Alzheimer's disease patients. *Eur. Neurol.* **2000**, *43*, 155–160.

35. Giedraitis, V.; Sundelof, J.; Irizarry, M.C.; Garevik, N.; Hyman, B.T.; Wahlund, L.O.; Ingelsson, M.; Lannfelt, L. The normal equilibrium between CSF and plasma amyloid beta levels is disrupted in Alzheimer's disease. *Neurosci. Lett.* **2007**, *427*, 127–131.
36. De Vos, A.; Jacobs, D.; Struyfs, H.; Fransen, E.; Andersson, K.; Portelius, E.; Andreasson, U.; De Surgeloose, D.; Hernalsteen, D.; Sleegers, K.; et al. C-terminal neurogranin is increased in cerebrospinal fluid but unchanged in plasma in Alzheimer's disease. *Alzheimers Dement.* **2015**, *11*, 1461–1469.
37. Janelidze, S.; Stomrud, E.; Palmqvist, S.; Zetterberg, H.; van Westen, D.; Jeromin, A.; Song, L.; Hanlon, D.; Tan Hehir, C.A.; Baker, D.; et al. Plasma beta-amyloid in Alzheimer's disease and vascular disease. *Sci. Rep.* **2016**, *6*, 26801.
38. Lewczuk, P.; Ermann, N.; Andreasson, U.; Schultheis, C.; Podhorna, J.; Spitzer, P.; Maler, J.M.; Kornhuber, J.; Blennow, K.; Zetterberg, H. Plasma neurofilament light as a potential biomarker of neurodegeneration in Alzheimer's disease. *Alzheimers Res. Ther.* **2018**, *10*, 71.
39. Palmqvist, S.; Janelidze, S.; Stomrud, E.; Zetterberg, H.; Karl, J.; Zink, K.; Bittner, T.; Mattsson, N.; Eichenlaub, U.; Blennow, K.; et al. Performance of Fully Automated Plasma Assays as Screening Tests for Alzheimer Disease-Related beta-Amyloid Status. *JAMA Neurol.* **2019**, doi:10.1001/jamaneurol.2019.1632.
40. Motter, R.; Vigo-Pelfrey, C.; Kholodenko, D.; Barbour, R.; Johnson-Wood, K.; Galasko, D.; Chang, L.; Miller, B.; Clark, C.; Green, R.; et al. Reduction of β-amyloid peptide42 in the cerebrospinal fluid of patients with Alzheimer's disease. *Ann. Neurol.***1995**, *38*, 643–648.
41. Riemenschneider, M.; Schmolke, M.; Lautenschlager, N.; Guder, W.G.; Vanderstichele, H.; Vanmechelen, E.; Kurz, A. Cerebrospinal beta-amyloid (1–42) in early Alzheimer's disease: Association with apolipoprotein E genotype and cognitive decline. *Neurosci. Lett.* **2000**, *284*, 85–88.
42. Clark, C.M.; Xie, S.; Chittams, J.; Ewbank, D.; Peskind, E.; Galasko, D.; Morris, J.C.; McKeel, D.W. Jr; Farlow, M.; Weitlauf, S.L.; et al. Cerebrospinal Fluid Tau and β-Amyloid: How Well Do These Biomarkers Reflect Autopsy-Confirmed Dementia Diagnoses? *Arch. Neurol.* **2003**, *60*, 1696–1702.
43. Thorsell, A.; Bjerke, M.; Gobom, J.; Brunhage, E.; Vanmechelen, E.; Andreasen, N.; Hansson, O.; Minthon, L.; Zetterberg, H.; Blennow, K. Neurogranin in cerebrospinal fluid as a marker of synaptic degeneration in Alzheimer's disease. *Brain Res.* **2010**, *1362*, 13–22.
44. Zetterberg, H.; Wilson, D.; Andreasson, U.; Minthon, L.; Blennow, K.; Randall, J.; Hansson, O. Plasma tau levels in Alzheimer's disease. *Alzheimers Res. Ther.* **2013**, *5*, 9.
45. Antonell, A.; Mansilla, A.; Rami, L.; Llado, A.; Iranzo, A.; Olives, J.; Balasa, M.; Sanchez-Valle, R.; Molinuevo, J.L. Cerebrospinal fluid level of YKL-40 protein in preclinical and prodromal Alzheimer's disease. *J. Alzheimers Dis.* **2014**, *42*, 901–908.
46. Kester, M.I.; Teunissen, C.E.; Crimmins, D.L.; Herries, E.M.; Ladenson, J.H.; Scheltens, P.; van der Flier, W.M.; Morris, J.C.; Holtzman, D.M.; Fagan, A.M. Neurogranin as a Cerebrospinal Fluid Biomarker for Synaptic Loss in Symptomatic Alzheimer Disease. *JAMA Neurol.* **2015**, *72*, 1275–1280.
47. Mattsson, N.; Zetterberg, H.; Janelidze, S.; Insel, P.S.; Andreasson, U.; Stomrud, E.; Palmqvist, S.; Baker, D.; Tan Hehir, C.A.; Jeromin, A.; et al. Plasma tau in Alzheimer disease. *Neurology* **2016**, *87*, 1827–1835.
48. Mattsson, N.; Andreasson, U.; Zetterberg, H.; Blennow, K.; Alzheimer's Disease Neuroimaging, I. Association of Plasma Neurofilament Light With Neurodegeneration in Patients With Alzheimer Disease. *JAMA Neurol.* **2017**, *74*, 557–566.
49. Wang, S.; Zhang, J.; Pan, T.; for Alzheimer's Disease Neuroimaging, I. APOE epsilon4 is associated with higher levels of CSF SNAP-25 in prodromal Alzheimer's disease. *Neurosci. Lett.* **2018**, *685*, 109–113.
50. Wang, L.; Alzheimer's Disease Neuroimaging, I. Association of cerebrospinal fluid Neurogranin with Alzheimer's disease. *Aging Clin. Exp. Res.* **2019**, *31*, 185–191.
51. Bateman, R.J.; Xiong, C.; Benzinger, T.L.; Fagan, A.M.; Goate, A.; Fox, N.C.; Marcus, D.S.; Cairns, N.J.; Xie, X.; Blazey, T.M.; et al. Clinical and biomarker changes in dominantly inherited Alzheimer's disease. *N Engl. J. Med.* **2012**, *367*, 795–804.
52. Niemantsverdriet, E.; Ottoy, J.; Somers, C.; De Roeck, E.; Struyfs, H.; Soetewey, F.; Verhaeghe, J.; Van den Bossche, T.; Van Mossevelde, S.; Goeman, J.; et al. The Cerebrospinal Fluid Abeta1-42/Abeta1-40 Ratio Improves Concordance with Amyloid-PET for Diagnosing Alzheimer's Disease in a Clinical Setting. *J. Alzheimers Dis.* **2017**, *60*, 561–576.

53. Lehmann, S.; Delaby, C.; Boursier, G.; Catteau, C.; Ginestet, N.; Tiers, L.; Maceski, A.; Navucet, S.; Paquet, C.; Dumurgier, J.; et al. Relevance of Abeta42/40 Ratio for Detection of Alzheimer Disease Pathology in Clinical Routine: The PLMR Scale. *Front. Aging Neurosci.* **2018**, *10*, 138.
54. Zetterberg, H. Plasma amyloid beta-quo vadis? *Neurobiol. Aging* **2015**, *36*, 2671–2673.
55. Zhao, Q.; Liu, M.; Ha, L.; Zhou, Y.; Alzheimer's Disease Neuroimaging, I. Quantitative (18)F-AV1451 Brain Tau PET Imaging in Cognitively Normal Older Adults, Mild Cognitive Impairment, and Alzheimer's Disease Patients. *Front. Neurol.* **2019**, *10*, 486.
56. Abu-Rumeileh, S.; Steinacker, P.; Polischi, B.; Mammana, A.; Bartoletti-Stella, A.; Oeckl, P.; Baiardi, S.; Zenesini, C.; Huss, A.; Cortelli, P.; et al. CSF biomarkers of neuroinflammation in distinct forms and subtypes of neurodegenerative dementia. *Alzheimers Res. Ther.* **2019**, *12*, 2.
57. Rosengren, L.E.; Karlsson, J.-E.; Karlsson, J.-O.; Persson, L.I.; Wikkelsø, C. Patients with Amyotrophic Lateral Sclerosis and Other Neurodegenerative Diseases Have Increased Levels of Neurofilament Protein in CSF. *J. Neurochem.* **1996**, *67*, 2013–2018.
58. Hellwig, K.; Kvartsberg, H.; Portelius, E.; Andreasson, U.; Oberstein, T.J.; Lewczuk, P.; Blennow, K.; Kornhuber, J.; Maler, J.M.; Zetterberg, H.; et al. Neurogranin and YKL-40: Independent markers of synaptic degeneration and neuroinflammation in Alzheimer's disease. *Alzheimers Res. Ther.* **2015**, *7*, 74.
59. Kvartsberg, H.; Portelius, E.; Andreasson, U.; Brinkmalm, G.; Hellwig, K.; Lelental, N.; Kornhuber, J.; Hansson, O.; Minthon, L.; Spitzer, P.; et al. Characterization of the postsynaptic protein neurogranin in paired cerebrospinal fluid and plasma samples from Alzheimer's disease patients and healthy controls. *Alzheimers Res. Ther.* **2015**, *7*, 40.
60. Brinkmalm, A.; Brinkmalm, G.; Honer, W.G.; Frölich, L.; Hausner, L.; Minthon, L.; Hansson, O.; Wallin, A.; Zetterberg, H.; Blennow, K.; et al. SNAP-25 is a promising novel cerebrospinal fluid biomarker for synapse degeneration in Alzheimer's disease. *Mol. Neurodegener.* **2014**, *9*, 53.
61. Zhang, H.; Therriault, J.; Kang, M.S.; Ng, K.P.; Pascoal, T.A.; Rosa-Neto, P.; Gauthier, S.; Alzheimer's Disease Neuroimaging, I. Cerebrospinal fluid synaptosomal-associated protein 25 is a key player in synaptic degeneration in mild cognitive impairment and Alzheimer's disease. *Alzheimers Res. Ther.* **2018**, *10*, 80.
62. Ishiki, A.; Kamada, M.; Kawamura, Y.; Terao, C.; Shimoda, F.; Tomita, N.; Arai, H.; Furukawa, K. Glial fibrillar acidic protein in the cerebrospinal fluid of Alzheimer's disease, dementia with Lewy bodies, and frontotemporal lobar degeneration. *J. Neurochem.* **2016**, *136*, 258–261.
63. Craig-Schapiro, R.; Perrin, R.J.; Roe, C.M.; Xiong, C.; Carter, D.; Cairns, N.J.; Mintun, M.A.; Peskind, E.R.; Li, G.; Galasko, D.R.; et al. YKL-40: A novel prognostic fluid biomarker for preclinical Alzheimer's disease. *Biol. Psychiatry* **2010**, *68*, 903–912.
64. Wennstrom, M.; Surova, Y.; Hall, S.; Nilsson, C.; Minthon, L.; Hansson, O.; Nielsen, H.M. The Inflammatory Marker YKL-40 Is Elevated in Cerebrospinal Fluid from Patients with Alzheimer's but Not Parkinson's Disease or Dementia with Lewy Bodies. *PLoS ONE* **2015**, *10*, e0135458.
65. Liu, L.; Herukka, S.K.; Minkeviciene, R.; van Groen, T.; Tanila, H. Longitudinal observation on CSF Abeta42 levels in young to middle-aged amyloid precursor protein/presenilin-1 doubly transgenic mice. *Neurobiol. Dis.* **2004**, *17*, 516–523.
66. Cho, S.M.; Lee, S.; Yang, S.H.; Kim, H.Y.; Lee, M.J.; Kim, H.V.; Kim, J.; Baek, S.; Yun, J.; Kim, D.; et al. Age-dependent inverse correlations in CSF and plasma amyloid-beta(1-42) concentrations prior to amyloid plaque deposition in the brain of 3xTg-AD mice. *Sci. Rep.* **2016**, *6*, 20185.
67. Bacioglu, M.; Maia, L.F.; Preische, O.; Schelle, J.; Apel, A.; Kaeser, S.A.; Schweighauser, M.; Eninger, T.; Lambert, M.; Pilotto, A.; et al. Neurofilament Light Chain in Blood and CSF as Marker of Disease Progression in Mouse Models and in Neurodegenerative Diseases. *Neuron* **2016**, *91*, 56–66.
68. Parent, M.J.; Zimmer, E.R.; Shin, M.; Kang, M.S.; Fonov, V.S.; Mathieu, A.; Aliaga, A.; Kostikov, A.; Do Carmo, S.; Dea, D.; et al. Multimodal Imaging in Rat Model Recapitulates Alzheimer's Disease Biomarkers Abnormalities. *J. Neurosci.* **2017**, *37*, 12263–12271.
69. Toledo, J.B.; Vanderstichele, H.; Figurski, M.; Aisen, P.S.; Petersen, R.C.; Weiner, M.W.; Jack, C.R. Jr.; Jagust, W.; Decarli, C.; Toga, A.W.; et al. Factors affecting Abeta plasma levels and their utility as biomarkers in ADNI. *Acta Neuropathol.* **2011**, *122*, 401–413.
70. Rembach, A.; Faux, N.G.; Watt, A.D.; Pertile, K.K.; Rumble, R.L.; Trounson, B.O.; Fowler, C.J.; Roberts, B.R.; Perez, K.A.; Li, Q.X.; et al. Changes in plasma amyloid beta in a longitudinal study of aging and Alzheimer's disease. *Alzheimers Dement.* **2014**, *10*, 53–61.

71. Verberk, I.M.W.; Slot, R.E.; Verfaillie, S.C.J.; Heijst, H.; Prins, N.D.; van Berckel, B.N.M.; Scheltens, P.; Teunissen, C.E.; van der Flier, W.M. Plasma Amyloid as Prescreener for the Earliest Alzheimer Pathological Changes. *Ann. Neurol.* **2018**, *84*, 648–658.
72. Nakamura, A.; Kaneko, N.; Villemagne, V.L.; Kato, T.; Doecke, J.; Dore, V.; Fowler, C.; Li, Q.X.; Martins, R.; Rowe, C.; et al. High performance plasma amyloid-beta biomarkers for Alzheimer's disease. *Nature* **2018**, *554*, 249–254.
73. Park, J.C.; Han, S.H.; Yi, D.; Byun, M.S.; Lee, J.H.; Jang, S.; Ko, K.; Jeon, S.Y.; Lee, Y.S.; Kim, Y.K.; et al. Plasma tau/amyloid-beta1-42 ratio predicts brain tau deposition and neurodegeneration in Alzheimer's disease. *Brain* **2019**, *142*, 771–786.
74. Zhou, W.; Zhang, J.; Ye, F.; Xu, G.; Su, H.; Su, Y.; Zhang, X.; Alzheimer's Disease Neuroimaging, I. Plasma neurofilament light chain levels in Alzheimer's disease. *Neurosci. Lett.* **2017**, *650*, 60–64.
75. Lin, Y.S.; Lee, W.J.; Wang, S.J.; Fuh, J.L. Levels of plasma neurofilament light chain and cognitive function in patients with Alzheimer or Parkinson disease. *Sci. Rep.* **2018**, *8*, 17368.
76. Oeckl, P.; Halbgebauer, S.; Anderl-Straub, S.; Steinacker, P.; Huss, A.M.; Neugebauer, H.; von Arnim, C.A.F.; Diehl-Schmid, J.; Grimmer, T.; Kornhuber, J.; et al. Glial Fibrillary Acidic Protein in Serum is Increased in Alzheimer's Disease and Correlates with Cognitive Impairment. *J. Alzheimers Dis.* **2019**, *67*, 481–488.
77. Villar-Pique, A.; Schmitz, M.; Hermann, P.; Goebel, S.; Bunck, T.; Varges, D.; Ferrer, I.; Riggert, J.; Llorens, F.; Zerr, I. Plasma YKL-40 in the spectrum of neurodegenerative dementia. *J. Neuroinflammation* **2019**, *16*, 145.
78. Li, L.; Jiang, Y.; Hu, W.; Tung, Y.C.; Dai, C.; Chu, D.; Gong, C.X.; Iqbal, K.; Liu, F. Pathological Alterations of Tau in Alzheimer's Disease and 3xTg-AD Mouse Brains. *Mol. Neurobiol.* **2019**, *56*, 6168–6183.
