# Supplementary material for: Utility of Animal Models to Understand Human Alzheimer’s Disease, Using the Mastermind Research Approach to Avoid Unnecessary Further Sacrifices of Animals
Source: Int J Mol Sci. 2020 Apr 30;21(9):3158. doi: 10.3390/ijms21093158 (PMC7247586; doi:10.3390/ijms21093158)
Supplement: Supplementary file 1 [file ijms-21-03158-s001.zip › Supplementary Material 3.pdf]

Supplementary Material 3

Table S2. Overview of literature researching for the selection of AD biomarkers in section 3.

Literature search tool: PubMed	
**Search Filters**	**Numbers of literatures found**
Cholinergic [Title/Abstract] AND Alzheimer [Title/Abstract]	1049
Tau [Title/Abstract] AND Alzheimer [Title/Abstract]	3173
Amyloid [Title/Abstract] AND Alzheimer [Title/Abstract]	7776
Reactive Gliosis [Title/Abstract] AND Alzheimer [Title/Abstract]	199
Oxidative stress [Title/Abstract] AND Alzheimer [Title/Abstract]	1116
Cerebrovascular [Title/Abstract] AND Alzheimer [Title/Abstract]	767
Neurodegeneration [Title/Abstract] AND Alzheimer [Title/Abstract]	1649
((Abeta1-42) AND CSF) AND Alzheimer	357
((Abeta1-42) AND plasma) AND Alzheimer	142
((Abeta1-40) AND CSF) AND Alzheimer	88
((Abeta1-40) AND plasma) AND Alzheimer	93
((P-tau) AND CSF) AND Alzheimer	566
((P-tau) AND plasma) AND Alzheimer	51
((T-tau) AND CSF) AND Alzheimer	520
((T-tau) AND plasma) AND Alzheimer	45
((neurofilament light) AND CSF) AND Alzheimer	114
((neurofilament light) AND plasma) AND Alzheimer	36
((neurogranin) AND CSF) AND Alzheimer	57
((neurogranin) AND plasma) AND Alzheimer	10
((SNAP-25) AND CSF) AND Alzheimer	6
((SNAP-25) AND plasma) AND Alzheimer	6
((GFAP) AND CSF) AND Alzheimer	25
((GFAP) AND plasma) AND Alzheimer	14
((YKL-40) AND CSF) AND Alzheimer	57
((YKL-40) AND plasma) AND Alzheimer	5
(PDAPP) AND Alzheimer	115
(Tg2576) AND Alzheimer	834
(APP23) AND Alzheimer	183
(APPPS1) AND Alzheimer	54
(APP/PS1) AND Alzheimer	1155
(3xTg-AD) AND Alzheimer	554
(TgF344-AD) AND Alzheimer	17
(McGill−R−Thy1−APP rat) AND Alzheimer	23
(Tg478_Tg1116_Tg11587_triple_homozygous rat) and Alzheimer	3
Ferrous_amyloid_buthionine (FAB) rat and Alzheimer	4
